# Modelling cholera in periodic environments

**DOI:** 10.1080/17513758.2014.896482

**Published:** 2014-03-10

**Authors:** Drew Posny, Jin Wang

**Affiliations:** ^a^ Department of Mathematics and Statistics, Old Dominion University, Norfolk, VA23529, USA

## Abstract

We propose a deterministic compartmental model for cholera dynamics in periodic environments. The model incorporates seasonal variation into a general formulation for the incidence (or, force of infection) and the pathogen concentration. The basic reproduction number of the periodic model is derived, based on which a careful analysis is conducted on the epidemic and endemic dynamics of cholera. Several specific examples are presented to demonstrate this general model, and numerical simulation results are used to validate the analytical prediction.

## 1. Introduction

Limited access to safe water and sanitation resources is common in developing countries, leaving them vulnerable to cholera outbreaks. Cholera is an intestinal infection caused by ingesting food or water contaminated with the bacterium *Vibrio cholerae*. If left untreated, an infected individual may become severely dehydrated and die within several days. In addition to prompt rehydration and medical treatment, proper sanitation facilities are needed to prevent infected individuals from shedding the bacteria back into the environment further fuelling the pathogen concentration and the persistence of the disease. Besides the transmission route based on environment–human interaction, the human-to-human direct transmission is also found important in shaping a cholera epidemic. A recent cholera outbreak in Zimbabwe, a land-locked country in Africa, during 2008–2009 underscores such a direct transmission pathway [12].

Numerous mathematical models have been published to analyse cholera outbreaks in an effort to better understand the complex disease transmission and determine adequate prevention and effective control strategies (see, for example, [6,7,9,11,12,16,17,19]). In particular, Wang and Liao [19] recently proposed a deterministic cholera model that incorporates general incidence and pathogen functions and that can unify many of the existing cholera models. These studies have certainly produced many useful results and have improved our understanding of cholera dynamics. One limitation of these models, however, is that most of them assumed that the model parameters are constant in time, meaning that the disease contact rate, recovery rate, pathogen growth rate, etc., all take fixed values independent of time. An exception, we note, is the work in [7] where, in addition to the main discussion on the autonomous cholera model, the author also conducted simple numerical tests to three scenarios with periodic coefficients. From the mathematical point of view, the constant parameter assumption has the advantage of simplifying the models and analysis, and facilitating the use of some well-known theory in autonomous dynamical systems.

On the other hand, environmental concerns, such as floods, droughts, temperatures and other climatic factors, are seasonal and could significantly affect cholera dynamics. For example, it has been observed that cholera is a seasonal disease in many endemic places and infection peaks often occur annually in the rainy or monsoon season [10,18]. Such filed observations underline the limitation of most (if not all) current mathematical cholera models and imply that mathematical insights into cholera seasonality has largely lagged behind. It is thus important for mathematical cholera studies to incorporate these seasonal factors to gain deeper quantitative understanding of the short- and long-term evolution of cholera dynamics, and to better predict and prevent future cholera outbreaks.

The objective of this paper is to propose a general cholera model in a periodic environment by extending the model proposed in [19] to include seasonal variations in the environment and the disease transmission pathways. In particular, the incidence (or, force of infection) and the rate of change for the pathogen concentration are subject to periodicity. Using the framework introduced in [20], we will analyse the basic reproduction number, *R*_0_, for this cholera model and establish that *R*_0_ is a sharp threshold for cholera dynamics in periodic environments: when *R*_0_<1, the disease-free equilibrium (DFE) is globally asymptotically stable, and the disease completely dies out; when *R*_0_>1, the system admits a positive periodic solution, and the disease is uniformly persistent. We mention that extinction and persistence results for some periodic epidemic systems are also discussed in [4,5,15,22].

The remainder of the paper is organized as follows. In Section 2, we introduce the periodic cholera model and state the necessary assumptions. In Section 3, the basic reproduction number is derived, followed by a global stability analysis of the disease-free equilibrium in Section 4. The existence and uniform persistence of an endemic periodic solution are analysed in Section 5. We then briefly study several specific cholera models in Section 6. Finally, conclusions are drawn in Section 7.

## 2. Mathematical model

Building on the cholera model in [19], we construct the following non-autonomous dynamical system to describe cholera dynamics in a periodic environment:

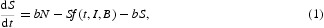

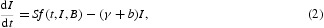

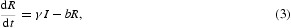

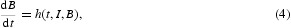

where *S, I, R* and *B* denote the susceptible population, infected population, recovered population and the concentration of vibrios in the contaminated water, respectively. The total population *N*=*S*+*I*+*R* is assumed to be a constant for all *t*≥0. The parameter *b* represents the natural human birth/death rate, and γ represents the rate of recovery from cholera. In this general model, the incidence function *f*(*t, I, B*) which determines the rate of new infection and the function *h*(*t, I, B*) which describes the rate of change for the pathogen in the environment are both differentiable and periodic in time with a common period ω. That is,

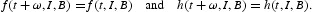

To make biological sense, we assume that the functions *f* and *h* satisfy the following conditions for all *t*≥0:

(A1) 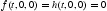.

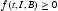
.

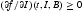
, 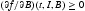, 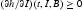, 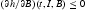.
*f*(*t, I, B*) and *h*(*t, I, B*) are both concave for any *t*≥0; i.e. the matrices

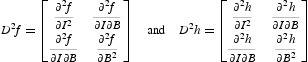

are negative semidefinite everywhere.

The assumption (A1) ensures that the model has a unique, constant disease-free equilibrium (DFE)

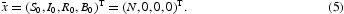

The assumption (A2) ensures a non-negative force of infection. The first two inequalities in (A3) state that the rate of new infection increases with both the infected population size and the pathogen concentration, and the third inequality states that increased human infection and, consequently, higher level of human contribution to the environmental vibrios, lead to higher growth rate for the pathogen. The last inequality in (A3) is based on experimental observation that the vibrios cannot sustain themselves in the environment in the absence of human contribution [13]; in other words, without the contribution from infected human population, the rate of change of the pathogen concentration would be negatively related to itself. The condition (A4) is based on saturation effect, a common assumption in epidemic models [19].

In addition, we assume that

(A5) 
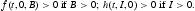

The first condition in (A5) implies that infection can start by the indirect transmission route alone; in other words, a positive bacterial concentration can lead to a positive incidence even if *I*=0 initially. The second condition in (A5) states that infected people will contribute to the growth of the vibrios in the environment (e.g. by shedding) even if *B*=0 initially.

Furthermore, we introduce an additional regulation on the profiles of the incidence and pathogen functions for small *I* and *B*. We assume that

(A6) There exists 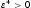 such that when 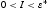, 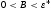,

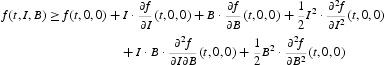

and

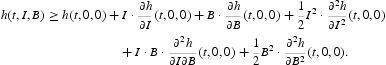

Here we make some comments on the assumption (A6). Based on the concavity of *f* (assumption A4), the surface of *f* is below its tangent plane everywhere. Meanwhile, since the matrix *D* ^2^ *f* is negative semidefinite, we have

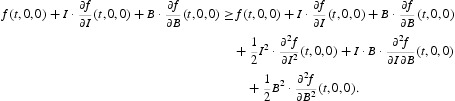

Thus, assumption (A6) essentially states that at least in a small neighbourhood of *I*=*B*=0, the surface of *f* lies below its tangent plane and above a concave tangent paraboloid. Similar reasoning holds for *h*.

Finally, we mention that many well-known cholera models, such as those in [7,9,12,17], all satisfy the above assumptions (A1)–(A6), though these models are based on autonomous dynamical systems. For example, the model in [12] has 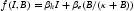 and 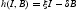. It is straightforward to verify that (A1)–(A6) hold; in particular, expanding *f*(*I, B*) at (0, 0) to second order yields 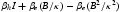, and it can be readily seen that *f*(*I, B*) satisfies (A6) as 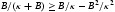 for all *B*>0. Similar verification can be done for the model in [7], where 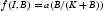, 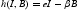, and the model in [17], where 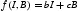, 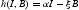. We will discuss in detail these models with periodic parameters in Section 6.

## 3. Basic reproduction number

A fundamental concept in epidemiology is the basic reproduction number, which measures the average number of secondary infections that occur when one infective is introduced into a completely susceptible host population. Following the standard next-generation matrix theory [8], we consider the subsystem of model (1)–(4) that is directly related to the infection:

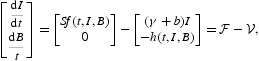

where 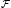 denotes the input rate of new infections and 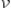 denotes the rate of transfer of individuals into or out of each population set. The next-generation matrix is defined as *F*(*t*)*V* ^−1^(*t*), where *F*(*t*) and *V*(*t*) are the Jacobian matrices given by

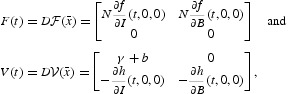

and where 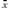 is the disease-free equilibrium of the model defined in Equation (5).

For a compartmental epidemiological model based on an *autonomous* system, the basic reproduction number is determined by the spectral radius of the next-generation matrix (which is independent of time) [8]. The definition of the basic reproduction number of a general non-autonomous model system, however, is still an open question. Bacaër and Guernaoui introduced *R*_0_ for periodic epidemic models (including ODE and PDE systems) as the spectral radius of an integral operator [2]; related work for some periodic ODE systems was also discussed in [1]. In addition, Wang and Zhao [20] extended the framework in [8] to include epidemiological models in periodic environments. They introduced the next infection operator *L* by

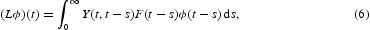

where *Y*(*t, s*), *t*≥*s*, is the evolution operator of the linear ω-periodic system 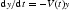 and φ(*t*), the initial distribution of infectious individuals, is ω-periodic and nonnegative. The basic reproduction number is then defined as the spectral radius of the next infection operator,

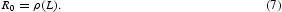

For our cholera model (1)–(4), the evolution operator can be easily determined by solving the system of differential equations 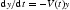 with the initial condition 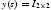; thus,

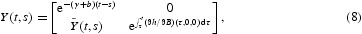

where

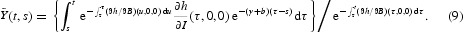

The basic reproduction number defined in Equation (7) can be numerically evaluated by using the methods presented in [1,14,20]. From [20], we immediately obtain the following result regarding the local stability of the DFE:

Theorem 1 Let *R*_0_ be defined as (7). Then the disease-free equilibrium of system (1)–(4) is locally asymptotically stable if *R*_0_<1, and unstable if *R*_0_>1.

## 4. Disease extinction

We proceed to investigate the global stability of the DFE for our cholera model, which will also provide a condition for the extinction of the disease. Consider the matrix function *F*(*t*)−*V*(*t*):

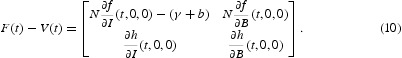

It can be easily verified that the above matrix function is continuous, cooperative, irreducible and ω-periodic. Let 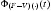 be the fundamental solution matrix of the linear ordinary differential system:

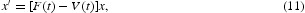

and 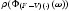 be the spectral radius of 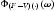.

From Lemma 2.1 in [22], we immediately obtain the following result:

lemma 2 Let 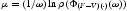. Then there exists a positive ω-periodic function *v*(*t*) such that 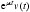 is a solution to Equation (11).

Now let us consider Equations (2) and (4) from our cholera model. It can be easily obtained, using assumption (*A*4), that

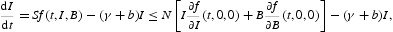

and

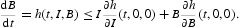

That is,

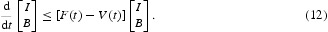

Meanwhile, based on Lemma 2, there exists *v*(*t*) such that

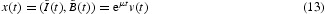

is a solution to Equation (11), with 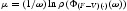. It follows from Equations (11) and (12) that

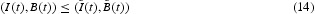

when *t* is large. From [20, Theorem 2.2], it is known that *R*_0_<1 if and only if 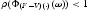. Therefore, μ<0. Then, given (13) and (14), it is clear that

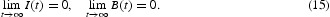

Next, we consider Equation (3) from our model. For any ε>0, there exists *T*>0 such that whenever *t*>*T*, we have

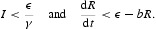

Thus, 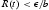 for *t*>*T*. Since ε>0 is arbitrary, it is clear that

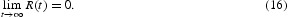

Finally, since the total population *N*=*S*+*I*+*R* is a constant, we have that

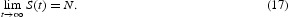

Hence, we have established the following result:

Theorem 3 If *R*_0_<1, then the disease-free equilibrium of model (1)–(4) is globally asymptotically stable, and 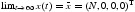 for any solution *x*(*t*) of system (1)–(4).

Theorem 3 shows that the disease will completely die out as long as *R*_0_<1. This further implies that reducing and keeping *R*_0_ below the unity would be sufficient to eradicate cholera infection even in a periodic environment. Similar result was established for the autonomous system in [19]; i.e. the cholera model with time-independent *f* and *h*.

## 5. Disease persistence

Now we consider the dynamics of the periodic model (1)–(4) when *R*_0_>1. For ease of discussion, let us omit Equation (3) from the system, since the total population *N* is fixed such that *R*=*N*−*S*−*I*. Define

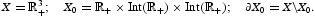

Let 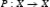 be the Poincaré map associated with models (1)–(4) such that 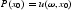 for all *x*_0_∈*X*, where *u*(*t, x*_0_) denotes the unique solution of the system with *u*(0, *x*_0_)=*x*_0_.

definition 4 The solutions of system (1)–(4) are said to be uniformly persistent if there exists some η>0 such that

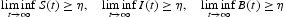

whenever *S*(0)>0, *I*(0)>0, and *B*(0)>0.

A more general definition of uniform persistence can be found in [24]. We now state the following theorem, the proof of which is inspired by the work of Zhang and Zhao [22].

Theorem 5 Let *R*_0_>1 and let (A1)–(A6) hold. Then the solutions of system (1)–(4) are uniformly persistent, and the system admits at least one positive ω-periodic solution.

*Proof* Set

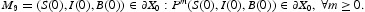

We first show that

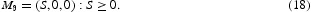

Clearly, 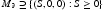. Consider any initial values 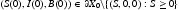. If *I*(0)=0 and *B*(0)>0, then *I*′(0)>0 by assumption (*A*5). Similarly, if *B*(0)=0 and *I*(0)>0, then *B*′(0)>0. Thus, it follows that 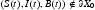 for 0<*t*≪1. This implies that 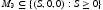, and hence, we have (18).

Now, let us consider the fixed point *M*_0_=(*N*, 0, 0) and define 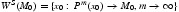. We show that

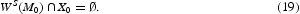

Based on the continuity of solutions with respect to the initial conditions, for any ε>0 and 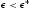, there exists δ>0 small enough such that for all 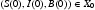 with 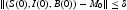, we have

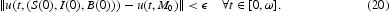

We claim that

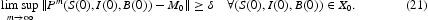

Suppose by contradiction; that is, we suppose 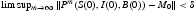 for some 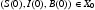. Without loss of generality, we assume that 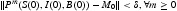. Thus,

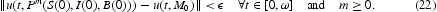

Moreover, for any *t*≥0, we can write 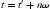 with 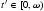 and *n* being the greatest integer less than or equal to *t*/ω. Then we obtain

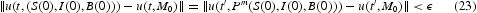

for any *t*≥0. Let 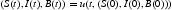. It follows that 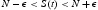, 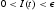 and 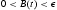. Note again that 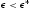. Then, based on assumptions (A1) and (A6), we have

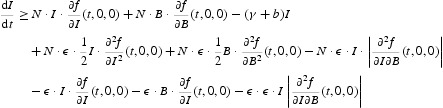

and

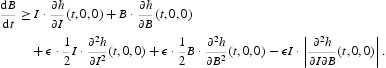

Hence, we obtain

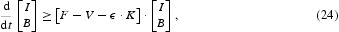

where *F*−*V* is given by (10) and

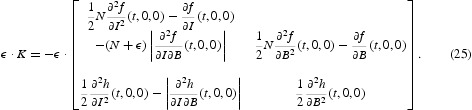

Again based on [20, Theorem 2.2], *R*_0_>1 if and only if 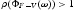. Thus, for ε>0 small enough, we have 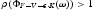. Using Lemma 2 and the comparison principle, we immediately obtain:

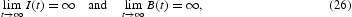

which is a contradiction. Hence, *M*_0_ is acyclic in *M*_∂_, and *P* is uniformly persistent with respect to 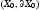, which implies the uniform persistence of the solutions to the original system [23]. Consequently, the Poincaré map *P* has a fixed point 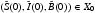, and it can be easily seen that 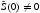. Thus, 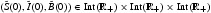 and 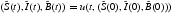 is a positive ω-periodic solution of the system.

## 6. Examples

In this section, we briefly discuss three different, and specific, cholera models in periodic environments. The models presented below are extended from recent work of Codeço [7], Mukandavire *et al*. [12], and Tien and Earn [17], respectively. We focus on simulating seasonal variations by incorporating periodic environment-to-human transmission rates and periodic rates of human contribution to the population of *V. cholerae* in the aquatic environment. We study the epidemic and endemic cholera dynamics of a hypothetical community with *N*=10, 000 as the (normalized) total population, and compute the basic reproduction number *R*_0_ for each model.

For comparison, we will also calculate the time-averaged reproduction number, denoted by [*R*_0_], for these cholera models. For any continuous periodic function *g*(*t*) with period ω, we may define its average as

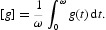

Keeping with this notation, we define the time-averaged matrices of *F*(*t*) and *V*(*t*) for the general cholera model (1)–(4) as the following, respectively,

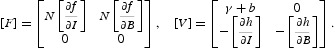

The time-averaged reproduction number of systems (1)–(4) is defined as the spectral radius of the time-averaged next-generation matrix [*F*][*V*]^−1^, and is given by

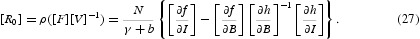

Based on Equation (27), the time-averaged reproduction number [*R*_0_] can be easily calculated for each of the three specific cholera models. It has been noted, however, that [*R*_0_] may overestimate or underestimate the infection risk for a non-autonomous epidemiological system [3,20]. Analytical estimates of the difference between *R*_0_ and [*R*_0_] for some periodic systems are also presented in [3]. Thus, it is of interest to compare the values of *R*_0_ and [*R*_0_] for the three cholera models under consideration.

Meanwhile, we conduct numerical simulation for each model with initial conditions *B*(0)=*R*(0)=0, *S*(0)=*N*−*I*(0), *I*(0)=1; that is, one infected individual enters an entirely susceptible community. For easy comparison, we use the same parameter setting for all the three models, and these parameter values are based on the cholera data published on the recent Zimbabwe cholera outbreak [12,21]. We present typical infection curves for both scenarios, *R*_0_<1 and *R*_0_>1, demonstrating disease extinction and disease persistence. Finally, in presenting each of these models, we keep the same notation for variables and parameters from the original autonomous model. We will clarify the different notation among the three extended models when necessary.

### 6.1 The model of Codeço with periodic parameters

The original model in [7] is now modified as

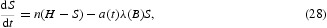

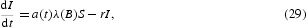

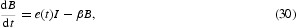

which includes seasonal oscillations of the rate of exposure to contaminated water, *a*(*t*), and the rate of human contribution to the population of the pathogen, *e*(*t*), that are both periodic functions of time with a common period, ω=365 days, or 1 year:

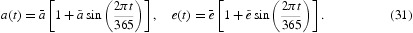

Here 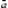 (or 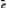) is the baseline value, or the time average, of *a*(*t*) (or *e*(*t*)), and *ã* (or *e˜*) denotes the (relative) amplitude of the seasonal oscillation in *a*(*t*) (or *e*(*t*)). To ensure both rates to be positive, we require that 0<*ã*<1, 0<*e˜*<1. In this model, *H* is the total population, 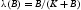 is the probability a susceptible person becomes infected with cholera, β=*mb*−*nb* represents the net death rate of vibrios, and only the environment-to-human transmission pathway is considered. The incidence is 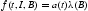 and the pathogen function is 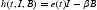. It is easily verified that the assumptions (A1)–(A6) hold for systems (28)–(30).

The disease-free equilibrium is given by 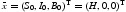. From the next-generation matrices

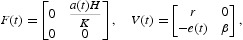

it follows that basic reproduction number of the time-averaged autonomous system, based on (27), is given by

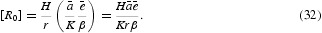

The evolution operator *Y*(*t, s*), defined in Equation (8), for this model is given by

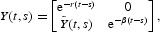

where

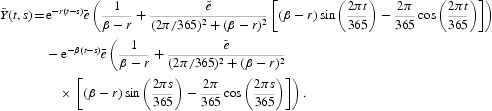

We then numerically evaluate the next infection operator (see Equation (6)) by

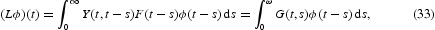

where

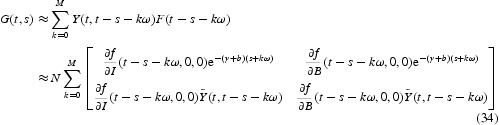

for some positive integer *M*. Thus, for models (28)–(30),

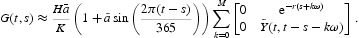

To compute the basic reproduction number *R*_0_, we reduce the operator eigenvalue problem to a matrix eigenvalue problem in the form of *A x*=λ *x*, where matrix *A* can be constructed by arranging the entries of the function *G*. The basic reproduction number *R*_0_ can then be approximated by numerically calculating the spectral radius of the matrix *A* [14]. Other methods for computing *R*_0_ also exist; for example, *R*_0_ can be numerically calculated by solving the equation *f*(*R*)=1, where *f*(*R*) is the dominant Floquet multiplier of 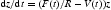 [1].

**Fig. 1.**
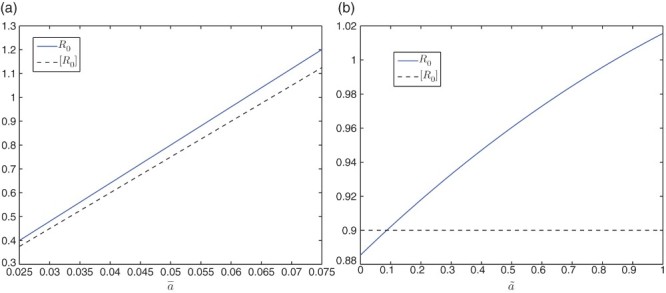
Plots of the periodic threshold of *R*_0_ for various *ā* and *ã*, respectively, in model 6.1. (a) *R*_0_=1 when *ā* ≈ 0.0625, and [*R*_0_]=1 when *ā* ≈ 0.0667; (b) *R*_0_=1 when *ã* ≈ 0.8407, and [*R*_0_]=0.90 for all *ã*.

**Fig. 4.**
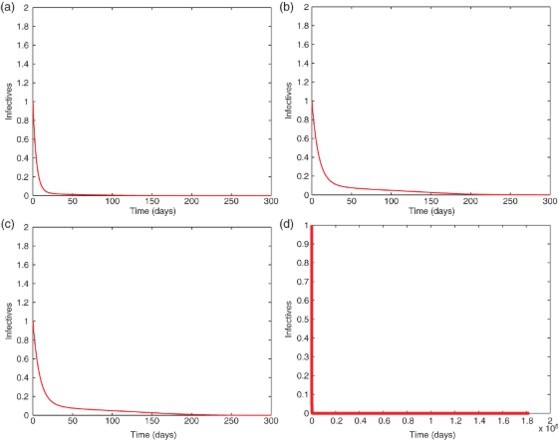
A typical infection curve for each model when *R*_0_<1, with initial condition *I*(0)=1. The solution quickly converges to the disease-free equilibrium with *I*_0_=0. (a) Model 6.1, (b) Model 6.2, (c) Model 6.3, (d) Model 6.3 in long term.

**Fig. 5.**
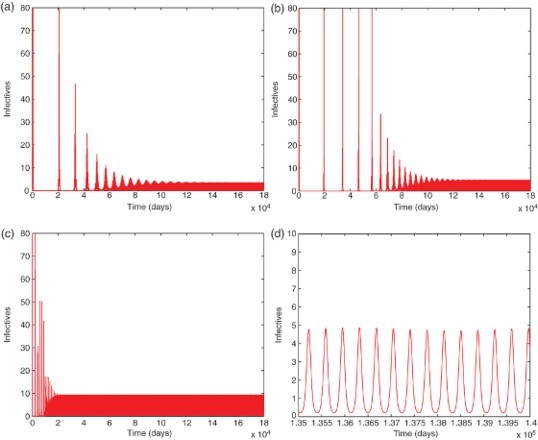
A typical infection curve for each model when *R*_0_>1, with initial condition *I*(0)=1. A periodic solution with ω=365 days forms after a long transient in each case. (a) Model 6.1, (b) Model 6.2, (c) Model 6.3, (d) Model 6.2 zoom-in.

We have conducted numerical simulation to this model, and computed the reproductive numbers *R*_0_ and [*R*_0_], for various values of *a*(*t*) and *e*(*t*). For illustration, we focus on the variation of *a*(*t*) here. In Figure 1(a) and 1(b), we vary 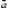 and *ã*, respectively, while keeping the values of other parameters fixed (see [12]): *H*=10, 000, *K*=10^6^, 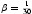, 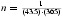, 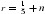, 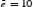, and *e˜*=0.5. In Figure 1(a), we see that *R*_0_=1 when 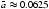, and [*R*_0_]=1 when 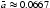. The value of *ã* is set as 0.5. It is clear that the time-averaged reproduction number underestimates the infection risk. Meanwhile, in Figure 1(b), we see that *R*_0_=1 when 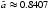, whereas [*R*_0_]=0.90 for all *ã*, again showing the inaccuracy of using [*R*_0_] for infection prediction. The value of 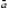 is set as 0.06 in this case. In addition, Figure 4(a) shows a typical infection curve of this model when *R*_0_<1, where we observe that the disease quickly dies out and the disease-free equilibrium is asymptotically stable. In contrast, Figure 5(a) is a typical infection curve of this model for *R*_0_>1, where the disease persists and there is a positive ω-periodic solution.

### 6.2 The model of Mukandavire *et al*. with periodic parameters

We extend the original model in [12] to a periodic environment based on the following differential equations:

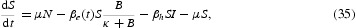

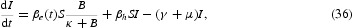

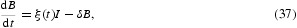

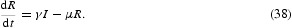

The two periodic parameters are defined as

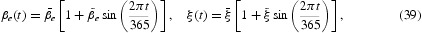

where β_*e*_(*t*) is the environment-to-human transmission rate and ξ(*t*) is the rate of contribution to *V. cholerae* in the aquatic environment. Though in different notation, β_*e*_(*t*) and ξ(*t*) have the same meaning as *a*(*t*) and *e*(*t*) in Equation (31). The incidence is 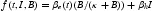 and the rate of change for the bacterial concentration is 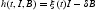. Both environment-to-human and human-to-human transmission pathways are included in this model; in particular, the environment-to-human transmission factor is based on a saturating form, which is the same as that in model (28)–(30), and the human-to-human transmission mode takes a bilinear form. It is clear that assumptions (A1)–(A6) hold for systems (35)–(38) as long as 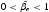 and 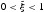.

The disease-free equilibrium is given by 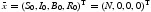. From the next generation matrices

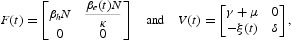

it follows that the time-averaged basic reproduction is

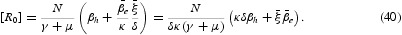

The evolution operator *Y*(*t, s*) is given by

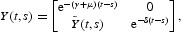

where

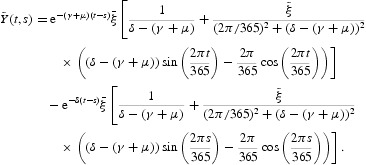

Thus, for this model,

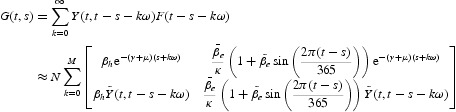

for some positive integer *M*. Using the function *G*(*t, s*), the basic reproduction number *R*_0_ can be numerically approximated by calculating the spectral radius of the corresponding matrix *A*.

**Fig. 2.**
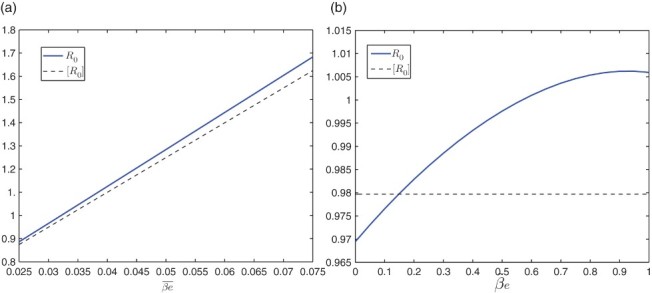
Plots of the periodic threshold of *R*_0_ for various β_*ē*_ and β_*e˜*_, respectively, in model 6.2. (a) *R*_0_=1 when β_*ē*_ ≈ 0.0321 and [*R*_0_]=1 when β_*ē*_ ≈ 0.0334; (b) *R*_0_=1 when β_*e˜*_ ≈ 0.5688 and [*R*_0_]=0.9797 for all β_*e˜*_.

In Figure 2(a) and 2(b), we vary 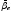 and 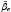, respectively, while keeping other parameters fixed: *N*=10, 000, κ=10^6^, 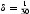, 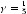, 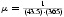, 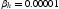, 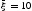, and 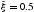. In Figure 2(a), we again observe that the curve of [*R*_0_] is below that of *R*_0_, and we note that *R*_0_=1 when 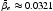. In Figure 2(b), we see that *R*_0_=1 when 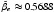 and 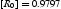 for all 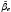. Note that 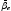 and 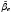 correspond to 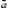 and *ã*, respectively, in Equation (31). Comparing the result in Figure 2(a) to that in Figure 1(a), we see that a lower value of the magnitude of the indirect transmission rate (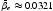 versus 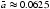) is needed to reach the threshold value *R*_0_=1 for the current model, due to the incorporation of the direct transmission mode. Similarly, we observe that the values of [*R*_0_] in Figure 2(a) and 2(b) are lower than those in Figure 1(a) and 1(b) for the same value of the parameter. In addition, Figure 4(b) is an infection curve when *R*_0_<1, and Figure 5(b) is an infection curve when *R*_0_>1, for the current model. We observe similar patterns as in Figures 4(a) and 5(a).

### 6.3 The model of Tien and Earn with periodic parameters

The original model in [17], where the pathogen concentration is denoted by *W* instead of *B*, is extended to a periodic environment in the form of

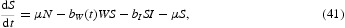

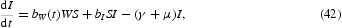

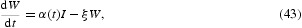

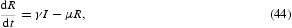

where

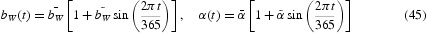

denote the water-to-person transmission rate and the shedding rate from infected individuals into the water, respectively. Here the time-periodic parameters *b*_*W*_(*t*) and α(*t*) play the same role as *a*(*t*) and *e*(*t*) in model (28)–(30), or β_*e*_(*t*) and ξ(*t*) in model (35)–(38). The incidence in the current model is 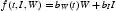 and the pathogen function is 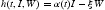. The dual-transmission pathways are included in this model by using bi-linear forms, however, no saturation effect was considered. It is straightforward to verify that assumptions (A1)–(A6) hold for systems (41)–(44) given that 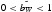, 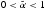.

Clearly, the DFE is given by 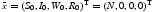. From the next-generation matrices

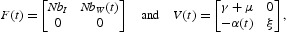



it follows that

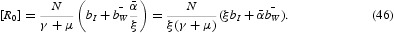

The evolution operator *Y*(*t, s*) is given by

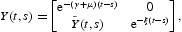

where

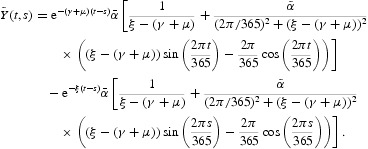

Thus, for models (41)–(44),

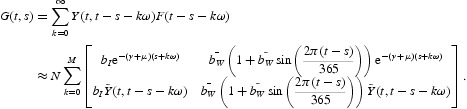

**Fig. 3.**
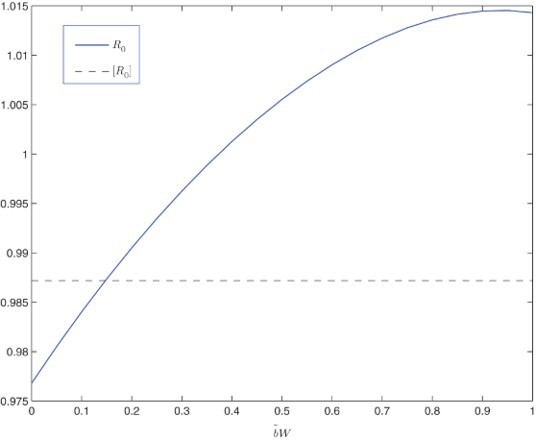
Plot of the periodic threshold of *R*_0_ for various *b*_*W˜*_ in model 6.3. *R*_0_=1 when *b*_*W˜*_ ≈ 0.3706 and [*R*_0_]=0.9872 for all *b*_*W˜*_.

We have conducted similar numerical simulations as before and calculated the two reproduction numbers. In presenting the results of *R*_0_, we could, in principle, vary 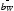 while keeping other parameters fixed. However, due to the bilinear form of the indirect transmission mode employed in the current model (and due to the very high value of *W*), the meaningful values of 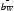 are several magnitudes smaller than those of 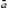 in Equation (31), or 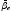 in Equation (39), making it impossible to compare the result with the other two models. Thus, we have chosen to present only the result of *R*_0_ (and [*R*_0_]) versus *b*_*W˜*_ in Figure 3. Values of the other parameters are: *N*=10, 000, 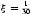, 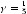, 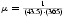, *b*_*I*_=0.00001, 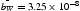, 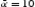, and 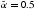. We see that *R*_0_=1 when 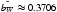 and 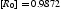 for all *b*_*W˜*_. The result shows similar pattern to that in Figure 2(b) as both models include dual transmission pathways. Figure 4(c) displays an infection curve when *R*_0_<1 for the current model, and Figure 5(c) shows an infection curve when *R*_0_>1.

Finally, from Figure 4(a)– 4(c), as expected, we see that when *R*_0_<1, the infected population *I* quickly decreases to zero and stays there forever (for example, see Figure 4(d) for the long-term behaviour of the model 6.3), showing that the disease dies out in each model. Indeed, similar patterns were observed for various initial conditions (not shown here), an evidence that the disease-free equilibrium is globally asymptotically stable for each model. Figure 5(a)–5(c) illustrates typical infection curves for the three models when *R*_0_>1. In this case, for each model, the disease persists and after a long, transient period, the infection approaches a positive ω-periodic solution. Figure 5(d) shows a zoomed-in picture for the model 6.2 where the periodic solution is highlighted and a period of ω=365 days (or 1 year) can be observed.

## 7. Conclusions

We have presented a general non-autonomous cholera model in a periodic environment. Seasonally variational factors have been incorporated into the incidence function *f* and the pathogen function *h*. Using the next infection operator introduced in [20], we have derived and computed the basic reproduction number *R*_0_ of our periodic cholera model, and have conducted a careful analysis on the epidemic and endemic dynamics. Our results have established *R*_0_ as a sharp threshold for cholera dynamics in periodic environments; i.e. disease completely dies out if *R*_0_<1 and uniformly persists if *R*_0_>1. The general analysis is demonstrated through three specific cholera models, and numerical simulation results are consistent with analytical predictions.

The complication of cholera modelling lies in that, on top of the multiple transmission pathways that involve both environment-to-human (or, indirect) and human-to-human (or, direct) routes, disease dynamics are also subject to strong seasonal variation. Thus, many different factors, ranging from ecological, environmental, societal, and climatic, need to be considered in constructing a more accurate mathematical model. We have incorporated periodicity into the general incidence and pathogen functions in our model, in order to represent these various seasonal oscillations in a generic manner. Although in the three specific examples presented in Section 6 we have focused on two periodic parameters (i.e. the rates of human–environment contact and human contribution to environmental vibrios) for the purposes of demonstration and easy comparison, one can easily incorporate periodicity into other model parameters, depending on the context of the modelling. In addition, similar analysis can be conducted to other cholera models (e.g. [9]), and the framework can be extended to model other water-borne infectious diseases, such as dysentery, typhoid fever, and campylobacteriosis.

This work was partially supported by the National Science Foundation under Grant Numbers 0813691 and 1245769. The authors are grateful to the two anonymous referees for their helpful comments to improve this paper.

## References

[CIT0001] (2007). *Approximation of the basic reproduction number *R*_0_ for vector-borne diseases with a periodic vector population*. Bull. Math. Biol.

[CIT0002] (2006). *The epidemic threshold of vector-borne disease with seasonality*. J. Math. Biol.

[CIT0003] (2007). *Growth rate and basic reproduction number for population models with a simple periodic factor*. Math. Biosci.

[CIT0004] (2011). *Threshold dynamics of a baillary dysentery model with seasonal fluctuations*. Disc. Cont. Dyn. Sys. Ser. B.

[CIT0005] (2012). *Global dynamics of an SEIRS epidemic model with periodic vaccination and seasonal contact rate*. Non. Anal.: Real World Appl.

[CIT0006] (2012). *On the global stability of a generalized cholera epidemiological model*. J. Biol. Dyn.

[CIT0007] (2001). *Endemic and epidemic dynamics of cholera: the role of the aquatic reservoir*. BMC Infect. Dis.

[CIT0008] (2002). *Reproduction numbers and sub-threshold endemic equilibria for compartmental models of disease transmission*. Math. Biosci.

[CIT0009] (2006). *Hyperinfectivity: a critical element in the ability of V. cholerae to cause epidemics*?.

[CIT0011] (2011). *Stability analysis and application of a mathematical cholera model*. Math. Biosci. Eng.

[CIT0012] *Estimating the reproductive numbers for the 2008–2009 cholera outbreaks in Zimbabwe*. Proc. Natl Acad. Sci. USA.

[CIT0013] (2009). *Cholera transmission: the host, pathogen and bacteriophage dynamics*. Nat. Rev.: Microbiology.

[CIT0015] (2012). *Persistence in seasonally forced epidemiological models*. J. Math. Biol.

[CIT0016] (2011). *Global dynamics of cholera models with differential infectivity*. Math. Biosci.

[CIT0017] (2010). *Multiple transmission pathways and disease dynamics in a waterborne pathogen model*. Bull. Math. Biol.

[CIT0019] (2012). *A generalized cholera model and epidemic-endemic analysis*. J. Biol. Dyn.

[CIT0020] (2008). Threshold dynamics for compartmental epidemic models in periodic environments. J. Dyn. Differential Equations.

[CIT0021] http://www.who.org.

[CIT0022] (2007). *A periodic epidemic model in a patchy environment*. J. Math. Anal. Appl.

[CIT0023] (2003). *Dynamical Systems in Population Biology*.

[CIT0024] (2001). *Uniform persistence in processes with application to nonautonomous competitive models*. J. Math. Anal. Appl.

